# Stellenwert der transpedikulären Biopsie bei Kypho- und Vertebroplastien von Wirbelkörperfrakturen: Eine Umfrage unter 250 Wirbelsäulenchirurgen

**DOI:** 10.1007/s00113-022-01210-y

**Published:** 2022-07-15

**Authors:** Georg Osterhoff, Denis Rappert, Max J. Scheyerer, Alexander C. Disch, Bernhard W. Ullrich, Ulrich A. Spiegl, Klaus J. Schnake, Arbeitsgruppe „Osteoporotische Frakturen“ und „Tumore“ der Sektion Wirbelsäule der Deutschen Gesellschaft für Orthopädie und Unfallchirurgie

**Affiliations:** 1 Klinik und Poliklinik für Orthopädie, Unfallchirurgie und Plastische Chirurgie, Universitätsklinikum Leipzig, Liebigstr. 20, 04103 Leipzig, Deutschland; 2 Interdisziplinäres Zentrum f. Wirbelsäulen- und Skoliosetherapie, Malteser Waldkrankenhaus St., Marien Rathsberger Str. 57, 91054 Erlangen, Deutschland; 3 Klinik und Poliklinik für Orthopädie und Unfallchirurgie, Medizinische Fakultät und Uniklinik Köln, Universität zu Köln, Kerpener Straße 62, 50937 Köln, Deutschland; 4 UniversitätsWirbelsäulenzentrum (UCSC), UniversitätsCentrum für Orthopädie, Unfall- & Plastische Chirurgie, Universitätsklinikum Carl Gustav Carus, TU Dresden, Fiedlerstraße 19, 01307 Dresden, Deutschland; 5 Klinik für Unfall- und Wiederherstellungschirurgie, BG Klinikum Bergmannstrost GgmbH Halle, Merseburger Straße 165, 06112 Halle (Saale), Deutschland; 6 Klinik für Unfall‑, Hand- und Wiederherstellungschirurgie, Universitätsklinikum Jena, Friedrich Schiller Universität Jena, 07747 Jena, Deutschland; 7 Klinik für Orthopädie und Unfallchirurgie, Klinikum Nürnberg Süd, Universitätsklinik der Paracelsus Medizinischen Privatuniversität, Nürnberg, Deutschland; 8 Deutsche Gesellschaft für Orthopädie und Unfallchirurgie (DGU), Berlin, Deutschland

**Keywords:** Tumor, Karzinom, Malignom, Metastase, Wirbelsäule, Diagnostik, Tumor, Cancer, Malignancy, Metastasis, Spine, Diagnostics

## Abstract

**Hintergrund:**

Transpedikuläre Zementaugmentationen sind eine etablierte Therapieoption in der Behandlung pathologischer Kompressionsfrakturen der Wirbelsäule. Neben der Osteoporose sind auch immer wieder metastasierte Grundleiden oder seltener ein primärer Knochentumor Ursache für Wirbelkompressionsfrakturen ohne adäquates Trauma.

**Ziel:**

Erstellung eines aktuellen Meinungsbildes unter Wirbelsäulenchirurgen in Deutschland, der Schweiz und Österreich zum Stellenwert der transpedikulären Biopsie während Kypho- und Vertebroplastien von Wirbelkörperfrakturen.

**Material und Methoden:**

Es wurde ein webbasierter UmfrageOnline®-Fragebogen mit 11 Fragen erstellt und an die E‑Mail-Verteiler der Deutschen Wirbelsäulengesellschaft (DWG), der Österreichischen Gesellschaft für Wirbelsäulenchirurgie (spine.at) und der Schweizerischen Gesellschaft für spinale Chirurgie (SGS) sowie an den E‑Mail-Verteiler der Sektion Wirbelsäule der Deutschen Gesellschaft für Orthopädie und Unfallchirurgie (DGOU) versendet.

**Ergebnisse:**

Von insgesamt kontaktierten 2675 Wirbelsäulenchirurgen beantworteten 250 (9,3 %) die Umfrage. Rund ein Drittel (29,8 %) der Befragten führt regelhaft bei jeder Kypho- oder Vertebroplastie eine transpedikuläre Biopsie durch. Genannte Gründe für eine Biopsie waren ein bildmorphologischer (79,7 %) oder anamnestischer Verdacht auf eine Tumorerkrankung (66,0 %) oder das Vorliegen einer solchen (71,4 %). Als Gründe gegen eine routinemäßige Biopsie wurden die damit verbundenen Kosten und die limitierte Aussagekraft der gewonnenen Biopsate genannt.

**Diskussion:**

Fast ein Drittel der befragten Wirbelsäulenchirurgen führt regelhaft bei jeder Kypho- oder Vertebroplastie eine transpedikuläre Biopsie durch. Fast alle Befragten führen Biopsien zumindest dann durch, wenn eine Tumorerkrankung bekannt ist bzw. aufgrund von Risikofaktoren vermutet wird. Zukünftige Studien müssen die Kosteneffizienz der transpedikulären Biopsie weiter abklären.

**Graphic abstract:**

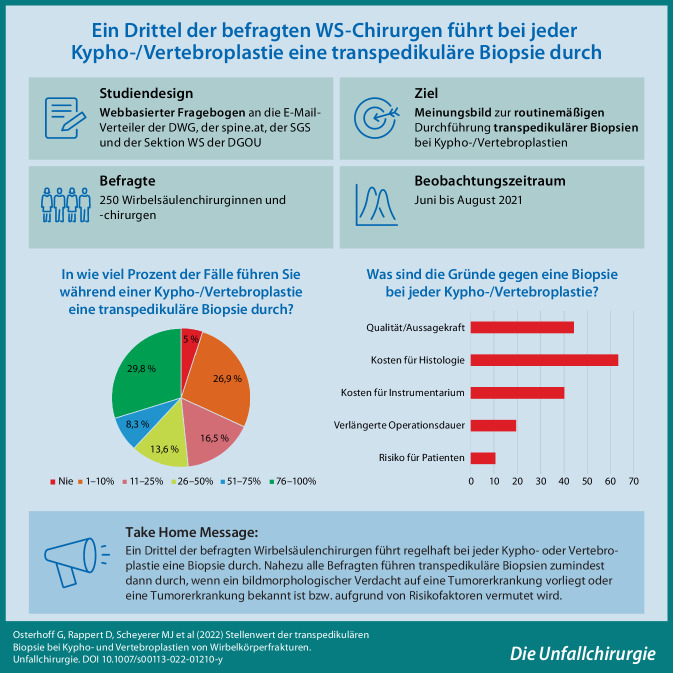

## Abstract (en)

**Background:**

Transpedicular cement augmentation is an established therapeutic option in the treatment of pathologic compression fractures of the spine. In addition to osteoporosis, underlying metastatic diseases or, more rarely, a primary bone tumor are recurrent causes of vertebral compression fractures without adequate trauma.

**Objective:**

To obtain a current opinion among spine surgeons in Germany, Switzerland, and Austria on the value of transpedicular biopsy during kyphoplasty and vertebroplasty of vertebral body fractures.

**Material and methods:**

A web-based (UmfrageOnline®) questionnaire with 11 questions was created and sent to the email distribution lists of the German Spine Society (DWG), the Austrian Society for Spine Surgery (spine.at), and the Swiss Society for Spinal Surgery (SGS), as well as to the email distribution list of the Spine Section of the German Society for Orthopedics and Trauma Surgery (DGOU).

**Results:**

Of a total of 2675 spine surgeons contacted 250 (9.3%) responded to the survey. Approximately one third (29.8%) of respondents regularly perform a transpedicular biopsy with each kyphoplasty or vertebroplasty. Reasons cited for biopsy were image morphology (79.7%) or history of suspected (66.0%) or present (71.4%) tumor disease. Reasons cited against routine biopsy were the associated costs and the limited informative value of the biopsies obtained.

**Discussion:**

Nearly one third of the spine surgeons surveyed regularly perform a transpedicular biopsy with each kyphoplasty or vertebroplasty. Almost all respondents perform biopsies at least when there is an imaging morphologic suspicion of tumor disease or tumor disease is known or suspected based on risk factors. Future studies need to further clarify the cost-effectiveness of transpedicular biopsy.

## Hintergrund und Fragestellung

Die mit Abstand häufigste Ursache für Wirbelkompressionsfrakturen ohne adäquates Trauma ist die Osteoporose [14]. Immer wieder kann jedoch auch ein metastasiertes Grundleiden, seltener ein primärer Knochentumor oder eine Spondylitis Ursache für solche Frakturen sein [4]. Die Wirbelsäule ist dabei eine der häufigsten Lokalisationen von Knochenmetastasen durch Primärtumoren anderer Lokalisation oder Manifestationen des multiplen Myeloms, die zusammen mit der Osteoporose ätiopathogenetisch die Gruppe der pathologischen Wirbelkörperfrakturen bilden [7, 9].

Während in den meisten Fällen metastatische und osteoporotische Frakturen anhand der Patientenanamnese und der bildgebenden Befunde unterschieden werden können, kann das Übersehen einer malignen Grunderkrankung für den Patienten mit in Folge Verzögerung oder sogar ein Ausbleiben der Tumortherapie fatal sein.

Kypho- oder Vertebroplastien haben sich als Therapieoption bei der Behandlung von Kompressionsfrakturen der Wirbelsäule über die letzten Jahrzehnte etabliert. Der transpedikuläre Zugang, der bei diesen Verfahren verwendet wird, ermöglicht bei allen Patienten problemlos eine Biopsie mit der Gewinnung von Gewebeanteilen des frakturierten Wirbelkörpers. Eine bei allen Patienten zusätzlich durchgeführte Biopsie, nimmt jedoch Zeit in Anspruch und ist mit erhöhten Kosten für die Biopsie-Utensilien und die histopathologische Aufarbeitung und Beurteilung verbunden [11].

Ziel dieser Umfrage unter Wirbelsäulenchirurgen in Deutschland, der Schweiz und Österreich war es, ein aktuelles Meinungsbild zum Stellenwert der transpedikulären Biopsie bei Kypho- und Vertebroplastien von Wirbelkörperfrakturen erstellen.

## Studiendesign und Methoden

Nach Initiative aus der Arbeitsgruppe Osteoporotische Frakturen und Arbeitsgruppe Tumoren der Sektion Wirbelsäule der Deutschen Gesellschaft für Orthopädie und Unfallchirurgie (DGOU) wurde mithilfe des Online-Tools UmfrageOnline® (www.umfrageonline.com) ein webbasierter Fragebogen mit 11 Fragen erstellt. Dieser umfasste Fragen zum klinischen Hintergrund und der Expertise der Befragten, zur Anzahl monatlich durchgeführter Kypho- oder Vertebroplastien, zur Häufigkeit durchgeführter Biopsien und zu Gründen für und gegen das Durchführen transpedikulärer Biopsien. Nach Freigabe durch die Wissenschaftskommissionen der Fachgesellschaften wurde der Fragebogen an die E‑Mail-Verteiler der Deutschen Wirbelsäulengesellschaft (DWG), der Österreichischen Gesellschaft für Wirbelsäulenchirurgie (spine.at) und der Schweizerischen Gesellschaft für spinale Chirurgie (SGS) sowie den E‑Mail-Verteiler der Sektion Wirbelsäule der DGOU versendet. Das Portal für die Beantwortung der Befragung war über 8 Wochen geöffnet.

Mit der Beantwortung des Fragebogens gaben die Teilnehmer ihr Einverständnis zur weiteren Verwendung der ausgewerteten Daten.

Nach Beendigung der Datenerhebung wurden die Daten in eine Statistiksoftware (SPSS V25; Fa. IBM, Armonk, NY, USA) exportiert, und es erfolgte eine deskriptive Datenanalyse.

In dieser Arbeit wird aus Gründen der besseren Lesbarkeit das generische Maskulinum verwendet. Weibliche und anderweitige Geschlechteridentitäten werden dabei ausdrücklich mitgemeint, soweit es für die Aussage erforderlich ist.

## Ergebnisse

### Hintergrund der Befragten

Insgesamt wurden 2675 Wirbelsäulenchirurgen angeschrieben. Die Umfrage wurde von 250 (9,3 %) Personen beantwortet, davon waren 119 (47,6 %) leitende Ärzte und Chefärzte, 93 (38,0 %) Oberärzte, 12 (4,9 %) angestellte Fachärzte, 6 (2,4 %) Ärzte in Weiterbildung und 15 (6,1 %) niedergelassene Ärzte mit vertraglicher Anbindung an ein Krankenhaus (5 Teilnehmer machten keine Angabe zur Position). Acht (3,3 %) der Befragten verfügten über ein Excellence-Zertifikat der DWG, 99 (40,2 %) über ein Master-Zertifikat und 55 (22,4 %) über ein Basis-Zertifikat, die übrigen 88 Befragten (34,1 %) waren nicht durch die DWG zertifiziert. Von den Befragten waren 56 (22,5 %) an Universitätskliniken, 62 (24,9 %) an nichtuniversitären Maximalversorgern, 59 (23,7 %) an Krankenhäusern der Schwerpunkt- und 52 (20,9 %) an Häusern der Grund- und Regelversorgung tätig. Weitere 21 (8 %) der Befragten waren an Privatkliniken oder anderen Fachkliniken beschäftigt (Tab. 1).

**Table 1:** 

Dienststellung	Krankenhaus					Total
	Universitätsklinik	Nichtuniversitärer Maximalversorger	Schwerpunktversorgung	Grund- und Regelversorgung	Privat- oder Fachklinik	
Leitende und Chefärzt*innen	17	30	33	30	9	119
Oberärzt*innen	28	21	22	16	6	93
Angestellte Fachärzt*innen	6	4	0	0	2	12
Ärzt*innen in Weiterbildung	4	1	0	1	0	6
Niedergelassene Ärzt*innen^a^	0	2	4	5	4	15
k. A.	1	4	0	0	0	5
Total	56	62	59	52	21	250

*k.* *A.* Keine Angabe

^a^Mit vertraglicher Anbindung an ein Krankenhaus

Dabei gaben 42 (16,8 %) der Befragten an, an einem durch die DWG zertifizierten „Wirbelsäulenzentrum der Maximalversorgung der DWG“ zu arbeiten, 34 (13,6 %) ordneten sich einem „Wirbelsäulenspezialzentrum der DWG“ und 17 (6,8 %) einer zertifizierten „Wirbelsäuleneinrichtung der DWG“ zu.

Die Anzahl der Patienten, bei denen pro Monat eine Kypho- oder Vertebroplastie (ggf. in Kombination mit einer perkutanen Instrumentierung) in den Kliniken der Befragten durchgeführt wird, wurde von 17,8 % mit > 10, von 26,4 % mit 5–10, von 28,5 % mit 3–5 und von 24,4 % mit einem bis 2 Patienten/Monat angegeben. Sieben (2,9 %) Befragte gaben an, keine Kyphoplastien/Vertebroplastien durchzuführen (Tab. 2).

**Table 2:** 

	In wie viel Prozent der Fälle führen Sie während einer Kypho- oder Vertebroplastie eine transpedikuläre Biopsie durch?							
	1–10 %	11–25 %	26–50 %	51–75 %	76–100 %	Nie	k. A.	Total
Master-Zertifikat	25 (25,3 %)	16 (16,2 %)	13 (13,1 %)	8 (8,1 %)	33 (33,3 %)	2 (2,0 %)	2 (2,0 %)	99 (100 %)
Excellence-Zertifikat	2 (25,0 %)	1 (12,5 %)	0 (0,0 %)	2 (25,0 %)	3 (37,5 %)	0 (0,0 %)	0 (0,0 %)	8 (100 %)
Basis-Zertifikat	13 (23,6 %)	13 (23,6 %)	5 (9,1 %)	6 (10,9 %)	14 (25,5 %)	4 (7,3 %)	0 (0,0 %)	55 (100 %)
Kein Zertifikat der DWG	25 (29,8 %)	10 (11,9 %)	15 (17,9 %)	4 (4,8 %)	22 (26,2 %)	6 (7,1 %)	2 (2,4 %)	84 (100 %)

Angegeben ist die Anzahl der Befragten, die die jeweils in den Spalten aufgeführte Antwort gewählt haben

*k.* *A.* Keine Angabe

Hier zeigte sich hinsichtlich der Häufigkeit durchgeführter Biopsien (0–50 % vs. > 50 %) kein statistischer Unterschied zwischen Chirurgen mit einem Master- oder Excellence-Zertifikat der DWG und solchen ohne oder mit Basis-Zertifikat (*p* = 0,178).

### Transpedikuläre Biopsien

**Figure 1:**
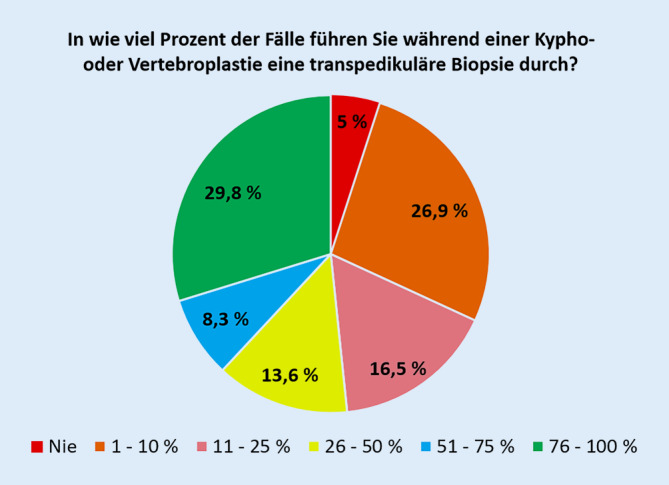

Nahezu alle (95 %) der Befragten führen zumindest manchmal (≥ 1 %) transpedikuläre Biopsien durch, 38,1 % tun dies in über der Hälfte aller Kypho‑/Vertebroplastien (Abb. 1).

**Figure 2:**
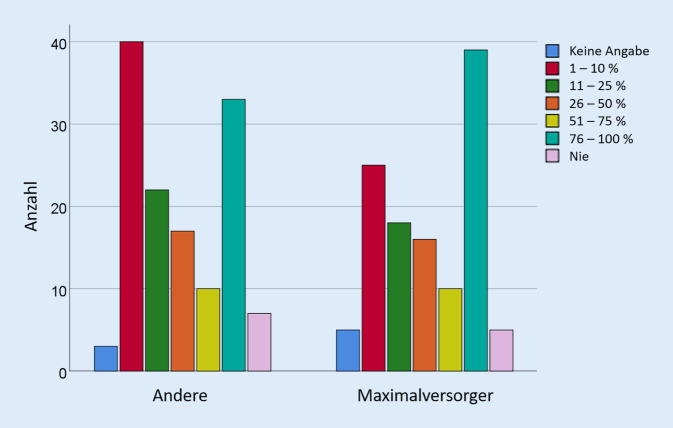

Dabei ist die Häufigkeit transpedikulärer Biopsien unabhängig von der Tatsache, ob sich die Befragten einem Krankenhaus der Maximalversorgung (universitär oder nichtuniversitär) zurechneten oder nicht (*p* = 0,615, Abb. 2).

Wird eine transpedikuläre Biopsie durchgeführt, so senden nur 2,6 % der Befragten meist oder immer intraoperativ Gewebe für eine histologische Schnellschnittdiagnostik mit ein, 81,6 % führen in diesem Rahmen nie eine Schnellschnittdiagnostik durch.

„Maximalversorger“ umfassen hier nichtuniversitäre und universitäre Maximalversorger. „Andere“ umfassen die Grund- und Regelversorger, Schwerpunktzentren und Fach- und Privatkliniken.

Falls eine transpedikuläre Biopsie mehrheitlich routinemäßig durchgeführt wird, so wurden folgende Gründe für das Durchführen einer solchen in absteigender Reihenfolge benannt (Mehrfachnennung möglich): 1. bildmorphologischer Verdacht auf eine Tumorerkrankung (79,7 %), 2. bekannte Tumorerkrankung (71,4 %), 3. wenn eine Tumorerkrankung anamnestisch oder aufgrund von Risikofaktoren vermutet wird (66,0 %), 4. intraoperativer Befund (24,9 %), 5. „sonstige Gründe“ (13,7 %) und 6. Patientenalter (8,3 %).

Das Vorliegen eines adäquaten Sturztraumas in der Anamnese wäre für 21,9 % der Befragten ein ausreichender Grund, keine Biopsie durchzuführen.

**Figure 3:**
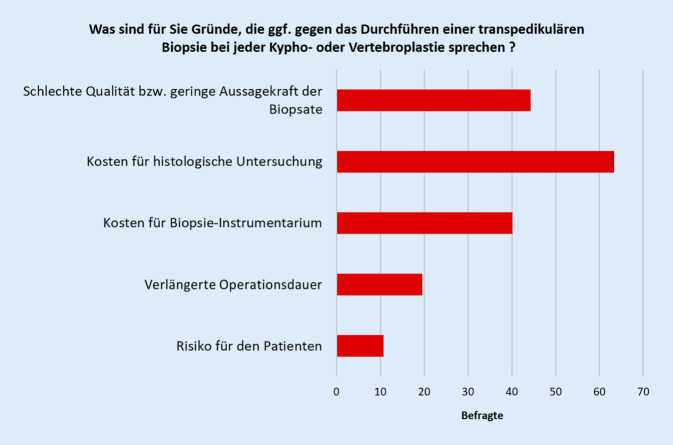

Als Gründe gegen die Durchführung einer routinemäßigen Biopsie wurden hauptsächlich die damit verbundenen Kosten sowie die „schlechte Qualität bzw. geringe Aussagekraft“ der gewonnenen Biopsate benannt (Abb. 3).

Eine Literaturrecherche der Autoren dieser Umfrage ergab, dass routinemäßige transpedikuläre Biopsien bei Kypho- oder Vertebroplastien von Kompressionsfrakturen der Wirbelsäule in 0,4 bis zu 6 % zur Diagnose bisher unbekannter Krebserkrankungen (primär und metastasiert) führen [6, 8, 11–13, 15, 16, 19, 20]. Im Bezug darauf wurden die Teilnehmer gefragt, ob sie angesichts dieser Ergebnisse den Stellenwert der Biopsien anders gewichten würden. Hier antworteten 54 (22,4 %), dass sie in Zukunft häufiger biopsieren würden und ein Befragter (0,4 %), dass er seltener biopsieren würde. Die große Mehrheit (186/77,2 %) würde auch angesichts dieser Zahlen genauso oft transpedikulär biopsieren wie bisher.

## Diskussion

Ziel dieser Umfrage unter Wirbelsäulenchirurgen in Deutschland, der Schweiz und Österreich war es, ein aktuelles Meinungsbild zum Stellenwert der transpedikulären Biopsie während Kypho- und Vertebroplastien von Wirbelkörperfrakturen zu erstellen. Transpedikuläre Kypho- und Vertebroplastien gehören zu den häufigsten Eingriffen an der Wirbelsäule und haben sich als Behandlungsoption für Wirbelkörperkompressionsfrakturen etabliert.

Die Umfrage erfolgte über die E‑Mail-Verteiler der führenden Fachgesellschaften für Wirbelsäulenchirurgie im deutschsprachigen Raum und erreichte 2675 Empfänger, sodass ein Kollektiv 250 erfahrener Wirbelsäulenchirurgen gewonnen werden konnte. Dies zeigt sich auch in dem hohen Anteil leitender Ärzte und durch die DWG zertifizierter Wirbelsäulenchirurgen unter den Befragten.

Nahezu alle Befragten führen transpedikuläre Biopsien zumindest manchmal durch, meist dann, wenn ein bildmorphologischer Verdacht auf eine Tumorerkrankung vorliegt oder eine Tumorerkrankung bekannt ist bzw. aufgrund von Risikofaktoren vermutet wird. Fast ein Drittel der Befragten führt eine Biopsie bei jeder Kypho- oder Vertebroplastie durch. Eine Schnellschnittdiagnostik wird dabei durch die wenigsten durchgeführt. Die wichtigsten genannten Gründe gegen die Durchführung einer routinemäßigen Biopsie sind die damit verbundenen Kosten und die Einschätzung, dass transpedikuläre Biopsie nur eine limitierte Aussagekraft haben. Der Grad der Zertifizierung der Operateure oder die Art des Krankenhauses (Maximalversorger vs. andere) scheint die Biopsieraten nicht zu beeinflussen.

In der Literatur wird die Prävalenz bisher unerkannter Primärtumoren oder metastasierter Erkrankungen bei Patienten mit Wirbelkörperkompressionsfrakturen in mehreren Studien untersucht [6, 8, 11–13, 16, 19, 20]. Routinemäßige transpedikuläre Biopsien während einer Kyphoplastie oder Vertebroplastie ergeben in 0,4–6 % der Fälle eine unerwartete maligne Diagnose, auch wenn die Definition von „unerwartet“ in den analysierten Studien variiert [6, 8, 11–13, 16, 19, 20].

Eine frühzeitige Diagnose kann zu einer früheren Tumorbehandlung und damit zu einer verbesserten Überlebenszeit und Restlebensqualität der Patienten führen. Ob dies auch für unerwartete Malignomdiagnosen nach Wirbelbiopsien zutrifft, kann aufgrund der schlechten Datenlage nicht abschließend beurteilt werden.

Dem potenziellen Vorteil einer früheren Diagnose stehen die Kosten für das Biopsie-Instrumentarium und die histopathologische Beurteilung gegenüber. Unter dem Gesichtspunkt, dass transpedikuläre Biopsien unabhängig von der Ausführung (fluoroskopisch, CT-gestützt oder offen durchgeführt) Diagnoseraten zwischen 85–90 % haben, ist von einer guten Kosteneffektivität routinemäßiger transpedikulärer Biopsien bei allen Kypho‑/Vertebroplastien auszugehen [10, 18]. Die Kosteneffektivität ist aufgrund höherer Sensitivität und Spezifität damit deutlich besser als bei anderen nichtinvasiven Methoden der Malignomfrüherkennung wie z. B. der Mammographie für die Brustkrebserkennung [2, 5]. Die hier vorgestellte Umfrage zeigt aber, dass der Qualität der transpedikulären Biopsie durch das befragte Kollektiv vornehmlich sehr erfahrener Wirbelsäulenchirurgen nur eingeschränkte Aussagekraft beigemessen wird.

Einige Autoren schlagen vor, Biopsien während einer Kypho‑/Vertebroplastie nur dann durchzuführen, wenn sich anamnestisch, klinisch oder bildmorphologisch der Verdacht auf eine Tumorerkrankung ergibt [8, 17]. Das deckt sich mit der Einschätzung von über einem Drittel der in unserer Studie Befragten.

Die Kombination aus Anamnese, körperlicher und laborchemischer Untersuchung sowie der Informationen verschiedener bildgebender Verfahren erlaubt eine Differenzierung zwischen Malignität und Osteoporose bereits mit hoher Genauigkeit [1, 4, 21].

Hier ist besonders anzumerken, dass ein Teil der im Rahmen dieser Studie Befragten nach Aufklärung über die mögliche Anzahl von unentdeckten Tumoren seine Meinung änderte und angesichts der Literatur mehr Biopsien durchführen würde. Das mag darauf hindeuten, dass es selbst unter erfahrenen Wirbelsäulenchirurgen an Wissen und Aufklärung fehlt. Es wäre möglich, dass bei besserer Aufklärung und transparenteren Kosten mehr Biopsien durchgeführt werden würden.

Umfragen haben bekannte Limitationen und können empirische klinische Daten zur Effizienz der transpedikulären Biopsie nicht ersetzen [3]. Die ausschließlich aus Wirbelsäulenchirurgen gewählte Stichprobe sowie der hohe Anteil von leitenden und Chefärzten schränken die Repräsentativität der Befragung ein. Angesichts der anonymen Befragung ist auch nicht auszuschließen, dass ein größerer Anteil der Antworten von Teilnehmern einiger weniger Kliniken stammt.

Mit einer Rücklaufquote von 9,5 % kann die Befragung nicht den Anspruch erheben, repräsentativ zu sein. Bei insgesamt 120 teilnehmenden Ärzten in Führungsposition und insgesamt 230 Fachärzten kann aber zumindest von einem Meinungsbild ausgegangen werden, welches die Versorgungsrealität im Ansatz widerspiegelt. Wichtiger noch, zeigt die Umfrage, dass es kein einheitliches Vorgehen gibt und hier Bedarf für weitere Forschung und Aufklärung besteht.

## Fazit für die Praxis

Fast ein Drittel der befragten Wirbelsäulenchirurgen führt regelhaft bei jeder Kypho- oder Vertebroplastie eine Biopsie durch. Nahezu alle Befragten führen transpedikuläre Biopsien zumindest dann durch, wenn ein bildmorphologischer Verdacht auf eine Tumorerkrankung vorliegt oder eine Tumorerkrankung bekannt ist bzw. aufgrund von Risikofaktoren vermutet wird. Genannte Gründe gegen die Durchführung einer routinemäßigen Biopsie sind die damit verbundenen Kosten und die vermutete limitierte Aussagekraft der transpedikulären Biopsie.
